# Clinical aspects of adherence to pharmacotherapy in Parkinson disease: A PRISMA-compliant systematic review

**DOI:** 10.1097/MD.0000000000010962

**Published:** 2018-06-18

**Authors:** Igor Straka, Michal Minár, Andrea Gažová, Peter Valkovič, Ján Kyselovič

**Affiliations:** a Second Department of Neurology, Faculty of Medicine Comenius University and University Hospital Bratislava; b Institute of Pharmacology and Clinical Pharmacology, Faculty of Medicine Comenius University; c Institute of Normal and Pathological Sciences, Slovak Academy of Sciences; d Department of Internal Medicine, Faculty of Medicine in Bratislava, Comenius University, Slovakia.

**Keywords:** adherence, influencing factors, Parkinson disease, pharmacotherapy, quality of life

## Abstract

**Background:**

Parkinson disease (PD) is the second most common neurodegenerative disease with various motor and nonmotor symptoms. Progressive course of PD requires frequent medication adjustments. Various combinations of drugs and dose regimens could be used to control symptoms. Thus, not surprisingly, adherence to pharmacotherapy is frequently suboptimal in these patients having negative effect on motor control and patient's quality of life.

**Methods:**

In this article, we offer up-to-date review of adherence in PD compared with other chronic conditions. In addition, we summarize factors influencing level of adherence, ways of measuring, and methods of adherence optimization. For the review of adherence in PD, a literature search was undertaken using PubMed database and relevant search terms. Articles were screened for suitability and data relevance.

**Results:**

PubMed and Scopus databases were systematically searched in 2016 and data extraction was a multistep process based on the PRISMA Guidelines.

**Conclusion:**

According to the recent data, sufficient control of motor symptoms and adequate quality of life are primary goals in the treatment of PD. Adherence to pharmacotherapy play a key role in this process, thus the medication should be tailored for each patient. In order to improve level of suboptimal adherence, these patients should have got recommended various dosing devices or alarms. Good communication with the patients and their relatives or caregivers is also essential.

## 1 Introduction

The patient's quality of life (QoL) has physical, mental/emotional, social, and economic aspects. Medicine-related problems of worsening QoL include issues related to medicine effectiveness, adverse reactions, and nonadherence to treatment.^[1]^ Adherence to medication is defined as the extent to which the patient's behavior agreed medical instructions of physician; it requires agreement between patient and physician. Compliance of therapy is necessary for satisfactory therapeutic effectiveness.^[2]^ Compliance is defined as a degree to which the patient's behavior matches to the doctor's recommendations. It is considered as a passive process of the patient. Concordance is a partnership between patient and physician based on an agreement. The patient either accepts, or does not accept proposed treatment, and patient's opinions should be respected.^[3,4]^

Nonadherence to treatment is one of the most common medicine-related problems in patients with chronic diseases. It increases costs because of the increase in hospital admissions, medical appointments, and other health care services.^[1]^ Adherence to treatment is influenced by several factors, such as mental state, sufficient information about the disease, good clinical control of condition, and some sociodemographic factors (e.g., supportive partner, good family background, and age). Race and gender do not play an important role contributing to the level of adherence. Main predictors of the low level of adherence to pharmacotherapy are summarized in the Table 1.^[5–23]^ Knowledge of the patient's risk for “non-adherence” is necessary in order to implement appropriate interventions for increased level of adherence.^[5,24]^

**Table 1:**
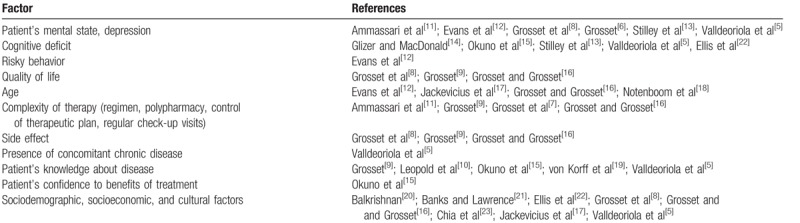
Factors influencing level of adherence.

Novel intervention for improvement of adherence is “adherence therapy” (compared with routine care). Adherence therapy is a cognitive-behavioral strategy based on patient beliefs’ impact on adherence. The principle of this approach is that patient and physician make choices collaboratively, and patients are more likely to cope with them because they are personally accepted and meaningful.^[25,26]^

## 2 Methods

We systematically reviewed publications focusing on factors associated with nonadherence to pharmacotherapy in PD and other selected chronic conditions.

### 2.1 Search methods

PubMed and Scopus databases were systematically searched in 2016 (updated October 2017).

For review of publications focusing on adherence in PD, the terms “Parkinson's disease” combined with “adherence,” “non-adherence,” “compliance,” “non-compliance” were used. We decided to compare adherence in PD patients to those with most frequent chronic disorder requiring life-long treatment.

For review of publications focusing on adherence in type 2 diabetes, the terms “type 2 diabetes” combined with “adherence,” “non-adherence,” “compliance,” “non-compliance” were used. For review of publications focusing on adherence in hypertension, the terms “hypertension” combined with “adherence,” “non-adherence,” “compliance,” “non-compliance” were used. For review of publications focusing on adherence in patients after myocardial infarction, the terms “myocardial infarction” combined with “adherence,” “non-adherence,” “compliance,” “non-compliance” were used.

### 2.2 Selection criteria

Titles and abstracts were reviewed for potential inclusion. Full-articles were obtained where abstracts appeared relevant. Studies meeting specific criteria were included:

(1) English or Slovak language;
(2) Full-text publication;
(3) Idiopathic PD population (defined by UK brain bank);
(4) Populations with selected chronic disorders (type 2 diabetes, hypertension, myocardial infarction);
(5) Presented data on medication adherence.

### 2.3 Exclusion criteria

We excluded studies if they were done on patients with diseases other than above-mentioned conditions and in other languages than in English or Slovak. In addition, editorials, case reports, and commentaries were excluded.

### 2.4 Quality assessment

Data extraction was a multistep process based on the PRISMA guidelines.^[27]^ For evaluation of the quality of nonrandomized studies, we used a modified version of the Newcastle–Ottawa scale.^[28]^ Studies were identified as having a low risk of bias (≥3 points) or a high risk of bias (<3 points). Summary of critical appraisal of included studies is in Table 2.^[6,7,9,11,12,16,21,22,29–38]^

**Table 2:**
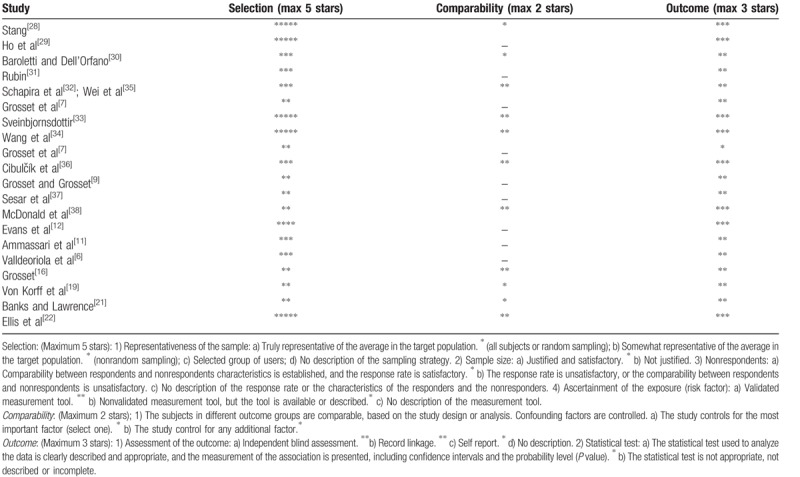
Summary of critical appraisal of included studies using the Newcastle–Ottawa scale for nonrandomized studies.^[28]^.

## 3 Adherence in chronic diseases

Chronic diseases are characterized by long-term medication use that is associated with reduced level of adherence to pharmacotherapy.

For example, frequently used basic pharmacotherapy after myocardial infarction is based upon combination of acetylsalicylic acid, statin, and beta-blocker. In a period of 1 month after hospital discharge, 34% of patients discontinued at least 1 of these 3 drugs, and 12% of patients completely stopped taking any of them.^[29,39]^ Primary nonadherence (de facto initial noncompliance with prescribed treatment) was associated with a significant increase of 1-year mortality. Secondary nonadherence (noncompliance with physician's instruction) was associated with increased mortality, frequent hospitalizations, and higher costs of treatment, compared with adherent patients.^[30]^

A retrospective analysis of 4783 patients taking antihypertensive medication showed that almost half of them discontinued treatment during a year – 48% of patients had at least 1 pause from using drugs and almost 95% of patients missed at least 1 dose during the year. That leads to inability to achieve a satisfactory blood pressure in patients with arterial hypertension, subsequently contributing to increased morbidity and mortality.^[40]^ Another study showed that less than two-thirds of patients did not achieve target level of blood pressure.^[41]^

In type 2 diabetes, a recent meta-analysis showed that treatment was sufficiently adhered by 36% to 85% of patients taking oral antidiabetic drugs, and by 60% to 80% of patients on insulin treatment.^[31,42]^

## 4 Adherence in Parkinson disease

Parkinson disease (PD) is a chronic progressive neurodegenerative disease that affects approximately 1% of persons older than 60 years. The QoL is significantly reduced in advanced stages of PD.^[43]^ The goal of therapy is to achieve good clinical outcome and to delay or alleviate long-term complications, for example, dyskinesias, motor, and nonmotor fluctuations, using a combinations of anti-Parkinson drugs in several daily doses. Moreover, control of motor symptoms in patients requires slow and careful titration of drugs, together with satisfactory adherence to daily medication regimen.^[44]^ Thus, it is not surprising that adherence to pharmacotherapy in PD patients is often suboptimal. Consequences resulting from reduced adherence to pharmacotherapy depend largely on the disease stage. In early-stage PD, occasionally missed doses do not usually clinically manifest. With the progression of PD, frequent omission of doses (or taking extra doses) may adversely affect motor control and QoL.^[2]^ Frequent missing of the dose leads to changes in pharmacokinetics of anti-Parkinson drugs, resulting to modified effectiveness of therapy.^[45]^

Dopaminergic therapy is associated with occurrence of side effects. Their profile depends on dose. Patients often report nausea, motor fluctuations, and hyperkinesias. Moreover, impulse control and repetitive behavior disorders and so-called dopamine dysregulation syndrome are seen at higher doses in more than 30% of PD subjects, especially in advanced stages.^[46,47]^

Omission of doses is more common than taking extra doses of drugs.^[6,7,32,48]^ Nonadherence is associated with progressive vanishing of therapeutic effect resulting in motor fluctuations and dyskinesias, and it is also connected with a higher risk of worsening symptoms.

Analyses of reduced adherence in PD showed that level of adherence correlates with younger age, complexity of therapeutic schedule, longer duration of disease, pill characteristics, mood disorders, reduced QoL, lack of knowledge about the disease, absence of a partner and insufficient family support, low income, and necessity to maintain a job.^[8,49]^ One-third of patients with PD have clinically significant depression. Depression is one of the most important factor of disability, reduced QoL, and satisfaction with medical care. Depressed patients have 3 times lower adherence to pharmacotherapy. In addition, nonadherence aggravates symptoms of depression, reduces QoL, and secondary leads to suboptimal use of medication. That encloses a vicious circle.^[49,50]^ Therefore, it is essential to identify depressive symptoms as soon as possible for effective management strategies (increase dopaminergic medication, add antidepressants).^[33,34]^

Higher level of adherence was observed in patients with a simple schedule of taking drug (the best results were with “once daily” pattern). Adverse effects of treatment also lead to reduced adherence and they are associated with arbitrary discontinuance of treatment regimen.^[8]^ Interestingly, in advanced stages of PD, an intentional omission of drug could prevent complications of levodopa therapy, for example, peak of dose dyskinesias. In contrast, sudden withdrawal of treatment could be fatal for patient and manifest as a neuroleptic malignant syndrome.^[49]^

Of importance, nonadherent patients have higher financial requirements, mostly caused by frequent therapy modification.^[35,49,51]^

In controlled setting of 1 large multicenter European study using electronic monitoring bottles (MEMS system),^[7]^ the overall median of adherence (doses taken/doses prescribed) was 97.7%, daily adherence was 86.2%, and time adherence was only 24.4%. More than 12% of patients took less than 80% of prescribed doses, defined as suboptimal adherence. The Unified PD Rating Scale motor score was significantly higher in patients with suboptimal adherence.^[7]^

A study assessing real-life adherence data from 219 outpatients with PD by means of patients’ questionnaires comprising of 4-item Morisky Medication Adherence Scale was done in Slovakia. A high level of adherence was observed in 52% of patients, moderate in 38% of patients, and low level in 9% . Omission, fear of adverse effects of drugs, and good clinical state were the most common causes of reduced adherence. Higher level of adherence was observed in patients taking medication once a day versus 2 and more times a day.^[36,53]^

Regularity in medication intake is very important in order to achieve control of symptoms.^[8,32,44]^ Day and time adherence are usually lower than total adherence. The analysis of studies of chronic diseases showed that total adherence is 71% ± 17% and time adherence is 59% ± 24%.^[53]^

Irregular medication intake is in conflict with the concept of continuous dopaminergic stimulation. Pulsatile dopaminergic stimulation of basal ganglia is probably the key reason for motor fluctuations. If severe and disabling hyperkinesia (interfering with activities of daily living) or painful off-state dystonia are present, there is a significant deterioration in the QoL of patients.^[9]^

## 5 Measuring level of adherence and methods of its optimization

Proper judgment of compliance and adherence is important for strategy and effectiveness of treatment.^[3]^ Each of the methods assessing level of adherence has advantages and disadvantages, as summarized in Table 3.

**Table 3:**
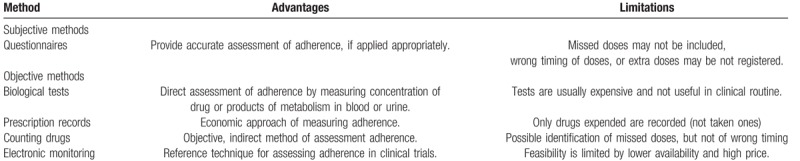
Methods for determining level of adherence.

In clinical practice, it is assessed mostly by questionnaires, in research settings mostly by electronic monitoring or counting drugs.^[54]^ For example, the questionnaire given to patients with PD revealed that 24.3% of patients referred omission of dose. But, using electronic monitoring (MEMS system), the omission was present in 51.3% of patients once a week, and in 20.5% of patients ≥3 times a week. Not taking the drug at scheduled times was reported by 73% of patients in questionnaire, but in 82.1% of patients in electronic monitoring (MEMS system).^[10]^ Although questionnaires are not sufficiently sensitive, they are highly specific for assessing adherence.

Patients taking 80% to 120% of prescribed doses of medication are considered to be optimally adherent; those taking less than 80% or more than 120% of prescribed doses are considered as suboptimally adherent.^[9,37]^

Basic interventions for adherence improvement include education of patient (with engagement of the relatives/caregivers into therapeutic process), improving communication between patient and clinician, dosage optimizing, and involvement of some tools to increase level of adherence (pill organizers, reminders, or other alarms). The most successful interventions are those involving combination of several methods.^[4,5]^

## 6 Practical recommendations to improve adherence to pharmacotherapy in patients with Parkinson disease

For good cooperation with the patient, a solid communication, an expression of concern with patient and satisfactory education (related to disease itself, the necessity of treatment, potential complications resulting from the disease, its treatment or nonadherence), all these are very essential. It is important to be assured that patient understood all given information. It is also appropriate to provide materials about the disease (information brochures). Engagement of other people into therapeutic process (spouse, other relatives, caregiver, psychotherapist, social worker, etc.)^[55,56]^ could also be helpful. Depression symptoms have to be taken into consideration among patients with low level of adherence.^[57]^

Various dosing devices, such as reminders, alarms set on cell phone, or wrist watch, are advisable tools for improving adherence. Using each method separately did not prove to be helpful in optimizing the adherence. That is why they should be combined with particular educational interventions.^[38,57]^

**Figure 1:**
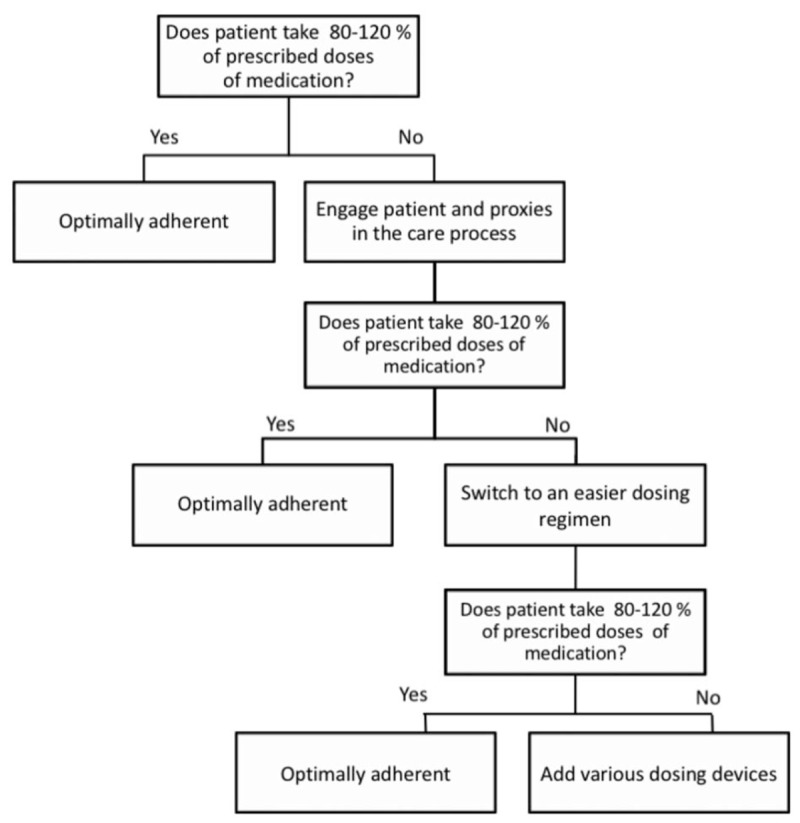
Algorithm to improve adherence to pharmacotherapy.^[37]^

Adherence may be also improved with easier dosing regimen, what could be achieved with controlled release dopamine receptor agonists (ropinirole, pramipexole) or that administrated by transdermal patch (rotigotine). Other option is referring patient to specialized movement disorders centers soon enough, so that they could be offered one of the advanced therapy options (levodopa/carbidopa intestinal infusion, continual subcutaneous apomorphine pump, or deep brain stimulation).^[37]^ Algorithm is shown on Fig. 1.

## 7 Conclusion

Level of adherence in PD is comparable to other chronic conditions. Sufficient control of motor symptoms and adequate QoL are primary goals in the treatment of PD, and the medication should be tailored for each patient. In order to improve level of suboptimal adherence, these patients should have got recommended various dosing devices or alarms. Good communication with the patients and their relatives or caregivers is also essential.

## Author contributions

**Conceptualization:** Peter Valkovic.

**Data curation:** Michal Minár.

**Project administration:** Igor Straka.

**Supervision:** Peter Valkovic, Ján Kyselovič.

**Visualization:** Andrea Gazova.
